# Antifungal Susceptibility of *Candida* Species Isolated from Horticulturists with Onychomycosis in Piauí, Brazil

**Published:** 2018-12

**Authors:** Mitra MOBIN, Maria Walderez SZESZS, Juliana Possatto TAKAHASHI, Marilena MARTINS, Daise Damaris Carnietto de HIPPÓLITO, Jhonatas Cley Santos PORTO, João Batista TELES, Sidney Gonçalo de LIMA, Marcia de Souza Carvalho MELHEM

**Affiliations:** 1. Research Laboratory, University Center UNINOVAFAPI, Teresina, Brazil; 2. Nucleus Mycology, Adolfo Lutz Institute, São Paulo, Brazil; 3. Science Center of Nature, Department of Chemistry, Federal University of Piauí, Teresina, Brazil

**Keywords:** *Candida*, Onychomycosis, Susceptibility, Matrix-assisted laser desorption/ionization mass spectrometry

## Abstract

**Background:**

We aimed to assess susceptibility pattern of *Candida* species isolated from horticulturists with onychomycosis to four antifungal drugs and to compare the effectiveness of conventional identification methods with matrix-assisted laser desorption/ionization mass spectrometry (MALDI-TOF MS).

**Methods:**

This study was conducted in a community garden located in Teresina, State of Piauí, Brazil, in the year 2014. The samples were identified through phenotypic methods and per MALDI-TOF MS, being used PCR as definitive identification test. The susceptibility pattern to four antifungal drugs was determined according to Clinical and Laboratory Standards Institute (CLSI).

**Results:**

Fourteen clinical isolates from seven different species were identified by the phenotypic method and by MALDI-TOF MS, with an observed concordance of 71.4% between the two methods. *C. albicans* (28.6%), *C. parapsilosis* (21.4%), *C. guilliermondii* and *C. metapsilosis* (both with 14.3%) were the most frequent species. With the exception of *C. krusei*, all species were sensitive to the tested antifungal.

**Conclusion:**

This is the first study of antifungal susceptibility of *Candida* in Piauí, Brazil. With the exception of *C. krusei*, no species showed resistance to the antifungal drugs used. This study suggests constants updates from the public databases used in MALDI-TOF MS to provide a rapid and accurate mycological diagnosis.

## Introduction

Onychomycosis is an infection resulting from the nail plate invasion by dermathophyte and, less frequently, by species of *Candida*, *Aspergillus*, *Scopulariopsis, Fusarium* and *Acremonium*. This disease is currently considered a public health problem because it affects about 10% of the world population and accounts for over 50% of all nail disorders (1).

*Candida* species are the second most common cause of onychomycosis, mainly affecting the fingernails. Although *C. albicans* is the most common etiological agent, other non-*albicans* species, such as *C. guilliermondii,* are currently gaining prominence in the medical field as nail mycosis agents (2, 3).

The susceptibility to antifungal drugs varies significantly among *Candida* species, as well as for the same species in different regions. Therefore, there is a constant need to determine antifungal susceptibility patterns of these organisms (3).

There is no report on susceptibility patterns of onychomycosis agents in Teresina- Piauí, Brazil. This research aimed to determine the susceptibility patterns of *Candida* species isolated from horticulturists with onychomycosis to four antifungal drugs and to compare the effectiveness of the conventional identification methods with MALDI-TOF MS.

## Materials and Methods

### Study Population and Location

This study was conducted in the year 2014 with horticulturists from a community garden located in Teresina, Piauí, Brazil, and approved by the Ethics in Research Committee of University Center UNINOVAFAPI, under CAAE 02070043000-10, obeying the Resolution 466/12. Participated in this research the horticulturists with at least 12 months of work, that presenting nail disorders and that sign the Informed Consent Form.

### Species collection and identification

Before initiating the procedure, the collection area was properly cleaned followed by antisepsis with 70% ethanol. Samples were collected during the dry and rainy seasons at intervals of 7 to 10 days. Posteriorly, the samples were transported to the Research Laboratory of the University Center UNINOVAFAPI – Piauí and to the Mycology Nucleus of the Adolfo Lutz Institute - São Paulo, for phenotypic identification and biochemical tests, respectively. The phenotypic identification was carried out according to to the identification keys described by (4–6). For the biochemical test was used the commercial system API^®^/ID32 (bioMérieux, Fr), following the manufacturer’s instructions.

The analysis per MALDI-TOF MS (VITEK MS, bio Mérieux, France) was carried out at the Mycology Nucleus of the Adolfo Lutz Institute - São Paulo, using a Microflex LT Mass spectrometer (Bruker Daltonik GmbH) (7). When there was a discrepancy between the results obtained by MALDI-TOF MS and by conventional identification, PCR was used with specific primers with starting sequences indicated in literature (8).

### Antifungal susceptibility test

In vitro antifungal susceptibility to four antifungal drugs – Fluconazole (Eurofarma, Brazil), Terbinafine (Genix Indústria Farmacêutica Ltda, Brazil), Itraconazole (Eurofarma, Brazil), and Amphotericin B (Sigma, S. Louis, EUA) – was determined through broth microdilution assay (9). The standard strains *C. parapsilosis* ATCC 22019 and *C. krusei* ATCC 6258 were used as controls (10). All tests were performed in triplicate.

The minimum inhibitory concentration (MIC) values were interpreted according to the new CLSI species-specific clinical breakpoints (CBPs) established for fluconazole against *Candida* species. For *Candida* species with no available CBPs, MIC was compared to the values available in literature. When it was not possible to assess MIC through breakpoint, epidemiological cutoff values (ECVs) were used (11).

### Statistical analysis

The entire statistical analysis was performed using the IBM SPSS statistics software, version 21.0 (Chicago, IL, USA). The variables were compared using Fisher’s exact test with significance level of 5%. Values of *P*<0.05 were considered significant for the analysis.

## Results

Fourteen clinical isolates from seven different species were identified as onychomycosis etiological agents in the horticulturists evaluated, being *C. albicans* with 28.6% (N=4) the most frequent species, followed by *C. parapsilosis* with 21.4% (N=3), *C. guilliermondii* and *C. metapsilosis* both with 14.3% (N=2), *C. africana, C. krusei* and *C. rugosa* both with 7.1% (N=1).

All yeasts were identified by the phenotypic method and MALDI-TOF MS, with a concordance of 71.4% between the two methods. The results obtained by the classical methodology differed from MALDI-TOF MS only for *C. albicans*, *C. parapsilosis* and *C. tropicalis*, identified by MALDI-TOF MS as *C. africana*, *C. metapsilosis,* and *C. rugosa,* respectively.

Concerning the susceptibility of *Candida* isolates identified in the present study to the four anti-fungal tested, only *C. krusei* showed resistance to fluconazole, with MIC of 64 μg/ml. *C. parapsilosis* (samples 11 and 30) presented low MIC values for all antifungal drugs analyzed while *C. guilliermondii* (sample 54) obtained the highest MIC values for all drugs, except fluconazole. The remaining yeasts presented very similar MIC values (Table 1).

**Table 1:** Antifungal drugs susceptibility in *Candida* species isolated from horticulturist with onychomycosis

***Samples***	***Species***	***Antifungals / MIC (μg/ml)***			
		FLC^*^	ITC^**^	TRB^***^	AMB^****^
9	*Candida krusei*	64	0.12	4	1
11	*Candida parapsilosis*	2	0.12	0.12	0.5
18	*Candida africana*	0.12	0.03	1	0.25
30	*Candida parapsilosis*	2	0.12	0.12	0.5
34	*Candida albicans*	2	0.015	0.5	0.25
43	*Candida metapsilosis*	4	0.12	0.25	0.5
44	*Candida albicans*	2	0.06	0.25	0.5
54	*Candida guilliermondii*	8	0.5	4	1
58	*Candida guilliermondii*	4	0.25	4	1
63	*Candida parapsilosis*	0.5	0.06	0.06	1
71	*Candida albicans*	0.12	0.015	0.25	0.25
89	*Candida albicans*	0.25	0.03	0.5	0.5
90	*Candida rugosa*	1	0.12	4	1
104	*Candida metapsilosis*	2	0.06	0.12	0.5
*Candida parapsilosis* ATCC- 22019		2	0.06	0.06	0.5
*Candida krusei* ATCC*- 6258*		64	0.12	4	1

Source: Laboratory of Research UNINOVAFAPI and Mycology Nucleus of the Adolfo Lutz Institute –SP

* Fluconazole

** Itraconazole

*** Terbinafine

**** Amphotericin B

## Discussion

Onychomycosis is the most common dermatomycosis in the world, affecting all populations and ages. *Candida* spp. is the second most frequent cause of nail mycosis in several geographical regions, affecting mainly fingernails. *C. albicans* and *C. parapsilosis* were the most frequently isolated species in this study (2,12, 13). There are no many studies addressing *C. guilliermondii*, the third most frequently found species in this study, as onychomycosis agent.

Making an accurate and rapid diagnosis is critical for a successful treatment of onychomycosis, but in many laboratories, this is not possible due to limited resources available. In terms of rapid, reliability and economy, MALDI-TOF MS is increasingly conquering space, not only in mycosis diagnosis but also in the diagnosis of pathologies caused by other types of microorganisms (12, 14).

MALDI-TOF MS analysis takes only few minutes and the execution protocols are simple, allowing high throughput of clinical samples. Species identification using this method depends on database consistency and on the number of spectra of different isolates belonging to the same species (15).

Yeast species identification was analyzed by the traditional method and MALDI-TOF MS and concluded that the latter promoted time saving and greater effectiveness (16). ITS region sequencing was used as reference method in performance analysis and verified 94% agreement between the identification results by MALDI-TOF MS and ITS and only 84.3% between classic analysis and the reference method.

MALDI-TOF MS cannot distinguish *C. africana* from *C. albicans*, requiring more precise molecular methods for such differentiation (12). Fortunately, the literature reports an effective method for distinguishing *C. albicans* from *C. africana*, based on the hyphal wall protein 1 (hwp1) gene in polymerase chain reaction (PCR) -based assay, where the hwp1 gene from *C. africana* contains 750bp and the typical *C. albicans* comprises 941bp (17).

In this study, the MALDI-TOF MS was able to determine critical species, such as *C. africana* and *C. metapsilosis*, indistinguishable within each complex according to phenotypic methods. The divergences between the results of this study and the other findings (12) can be justified by the inconsistency of the public databases, reaffirming the need for continuous updating of such databases (7,18).

In relation to the four antifungal drugs tested, itraconazole and terbinafine are used in skin mycosis treatment whereas amphotericin B is recommended for more severe cases of fungal infections (19). Fluconazole is the most commonly used agent for the prophylaxis and treatment of candidiasis, considering its low toxicity, high solubility and wide tissue distribution. Due to these pharmacological characteristics added to the emergence of azole-resistant *Candida* species, one study has synthesized, from changes in the structure of fluconazole, two compounds named aryl-1,2,4-triazole-3-yl analogue of fluconazole (ATTAF-1) and aryl-1,2,4-triazole-3-yl (thio) analogues of fluconazole-2 (ATTAF-2). These new azole compounds showed low toxicity in human cells and effective anti-*Candida* activity in vitro both alone and in combination with fluconazole, proving to be an effective alternative for the treatment of *Candida* spp. infections (20).

Onychomycosis by yeasts does not always respond well to topical treatment and systemic treatment is necessary in those cases. The drugs used in systemic treatment belong to two big pharmaceutical groups: allylamines and azole derivatives. Terbinafine is an allylamine effective in the treatment of onychomycosis by *Candida* species, mainly *C. parapsilosis* (21,2).

Unfortunately, studies on yeast susceptibility in onychomycosis cases are scarce in literature and data is usually reported together with yeasts that cause mucocutaneous, urinary and bloodstream infections. In this manner, the assessment of just onychomycosis strains is impaired.

With the exception of *C. krusei*, whose intrinsic resistance to fluconazole is already known, in this study no resistance was observed to other anti-fungal drugs. However, the MICs obtained for *C. guilliermondii* (case 54) against the four antifungal drugs tested must be highlighted, once they were the highest MICs obtained for a single isolate.

*C. guilliermondii* is currently a species that raises great interest and concern among medical doctors because of the emergence of strains with multi-resistance to azoles, terbinafine and amphotericin B. High values of fluconazole and terbinafine were already observed for a single isolate of this species (21, 22).

## Conclusion

This is the first study of antifungal susceptibility of *Candida* spp. in Piauí, Brazil. With the exception of *Candida krusei*, no species showed resistance to the four antifungal drugs used. This study suggests constants updates from the public databases used in MALDI-TOF MS to provide a rapid and accurate mycological diagnosis.

## Ethical considerations

Ethical issues (Including plagiarism, informed consent, misconduct, data fabrication and/or falsification, double publication and/or submission, redundancy, etc.) have been completely observed by the authors.
